# Effect of leisure activities on cognitive aging in older adults: A systematic review and meta-analysis

**DOI:** 10.3389/fpsyg.2022.1080740

**Published:** 2022-12-22

**Authors:** Xinxin Yang, Xin Yi Xu, Linlin Guo, Yuanyuan Zhang, Shan Shan Wang, Yan Li

**Affiliations:** ^1^School of Nursing, Hebei Medical University, Shijiazhuang, Hebei, China; ^2^Postdoctoral Research Station in Basic Medicine, Hebei Medical University, Shijiazhuang, Hebei, China; ^3^Centre for Gerontological Nursing, School of Nursing, The Hong Kong Polytechnic University, Hong Kong, China; ^4^School of Nursing and Health, Zhengzhou University, Zhengzhou, Henan, China; ^5^Neuroscience Research Center, Hebei Medical University, Shijiazhuang, Hebei, China; ^6^Hebei Key Laboratory of Neurodegenerative Disease Mechanism, Shijiazhuang, Hebei, China

**Keywords:** leisure activities, cognition, older adults, meta-analysis, meta-regression

## Abstract

Abnormal cognitive aging in older adults is a growing public health problem. Previous studies showed inconsistent results pertaining to the effects of leisure activities on cognitive function in older adults. We conducted a systematic review and meta-analysis of published observational longitudinal studies to examine and synthesize the effects of leisure activities on cognitive function in older adults. MEDLINE, PubMed, EMBASE, PsycINFO (Ovid), CINAHL (EBSCO), and Web of Science databases were searched from January 2012 to January 2022. Relative risks (RRs) with 95% confidence intervals (CIs) were pooled using random-effects meta-analysis. Most studies found that leisure activities had a positive effect on cognitive function in older adults. The pooled RR for the effect of leisure activity on cognitive function was 0.77 (95% CI: 0.72–0.81, *p* < 0.01). The effects of leisure activities on cognitive function varied by different cognitive statuses in older adults, with RRs ranging from 0.55 (95% CI: 0.37–0.83) to 1.07 (95% CI: 0.95–1.22). Meta-regression analysis showed that compared with studies with percentage of female ≥50%, studies with female participant percentage <50% had significantly increased RR (*p* = 0.01). Moreover, studies conducted in European and American countries had significantly lower RR (*p* = 0.019), compared with those conducted in Asian countries. Our study revealed different effects of various types of leisure activities on different cognitive statuses in older adults. To make innovative recommendations for promoting cognitive function in older adults, more detailed observational longitudinal studies investigating the effects of different types of leisure activities on different cognitive statuses in older adults are needed.

## 1. Introduction

Abnormal cognitive aging in older adults is a growing public health issue, moreover, increased life expectancy and population aging are expected to substantially increase the number of people with mild cognitive impairment (MCI) and dementia (Hu et al., 2017; Nichols et al., 2022). Globally, a large proportion of older adults are reportedly affected by MCI (Hu et al., 2017). Furthermore, previous studies have reported high rates of conversion from MCI to dementia (Zhang et al., 2021b). According to the World Health Organization, 2019, there were 55.2 million people with dementia globally, and the global cost of dementia was estimated at US$1.3 trillion in 2019 (World Health Organization, 2021). Dementia places a huge burden on patients, families, society, and especially the healthcare system (World Health Organization, 2021; Nichols et al., 2022). Over the past few decades, no effective treatment for dementia has been developed, not even a disease-modifying therapy (Liang et al., 2020). Therefore, some scholars have proposed that studies should focus more on dementia prevention than on dementia treatment (Fyfe, 2015). A recent study has reported that the self-management of modifiable risk factors can improve cognitive performance and reduce the risk of abnormal cognitive aging (Livingston et al., 2020). Thus, the search for modifiable preventive factors for abnormal cognitive aging has become increasingly urgent.

Leisure activities are defined as activities those in which individuals participate for enjoyment or wellbeing, these activities are independent from work or activities of daily living, which mainly include physical, cognitive, and social activities (Verghese et al., 2006; Wang et al., 2012; Mao et al., 2020; Zhang et al., 2021a). A study has shown that various intellectual, physical and social activities can produce cognitive enrichment effects to delay or alleviate cognitive decline in older adults (Hertzog et al., 2008). Participating in leisure activities is considered a promising direction for improving cognitive function in older adults. Regarding the relationship between activity engagement and cognitive performance in older adults, Bielak et al. proposed a “use it or lose it” theory (Bielak, 2010). But the relationship between leisure activity engagement and cognitive performance in older adults still needs further exploration.

Numerous studies have found positive associations of physical, cognitive, and social activities with cognitive function in older adults (Wang et al., 2012; Livingston et al., 2020). Effects of physical, cognitive and social activities on cognitive function in older adults appear to have common pathways, rather than specific mechanisms. They may increase cognitive reserve, reduce stress, and improve cardiovascular health in older adults to improve cognitive performance in older adults (Fratiglioni et al., 2004). A previous study concluded that physical activity is a protective factor of cognitive function in older adults, although there is no consensus on the effects of cognitive and social activities on cognitive function in older adults. Shin et al. (2021) reported that cognitive activity is a protective factor for cognitive function among older adults. In contrast, Anstey et al. (2008) found that intellectual–cultural activity is not related to the risk of dementia in older adults. The Lancet Commission on Dementia Prevention, Intervention, and Care reported that social activity can mitigate cognitive decline (Livingston et al., 2020). However, social activity was not included in the protective factors of cognitive function in older adults reported by WHO guidelines of Risk Reduction of Cognitive Decline and Dementia (World Health Organization, 2019). Therefore, a meta-analysis of published data on the effect of leisure activities on cognitive function in older adults is need to address the limitations and inconsistent evidence base of previous studies.

Moreover, most previous systematic reviews and meta-analyses have synthesized the effect of only a single type of leisure activity on cognitive function. For example, Sajeev et al. only synthesized the effect of cognitive activity on dementia (Sajeev et al., 2016), and Venegas-Sanabria et al. only investigated the effect of physical activity on cognitive impairment (Venegas-Sanabria et al., 2021). Previous studies have not compared the effects of different types of leisure activities on cognitive function. Meanwhile, most of the previous meta-analyses focused on interventional studies (Wollesen et al., 2020; Venegas-Sanabria et al., 2021). A systematic review and meta-analysis of observational longitudinal studies is needed to elucidate the details of how leisure activities are associated with the development of cognitive aging in older adults. Observational longitudinal studies can identify real-world conditions, and their results provide better external validity, and are more easily transferable to the general older adults with certain mobility. Therefore, we conducted a systematic review and meta-analysis of longitudinal studies to investigate the effect of leisure activities on cognitive function in older adults and compare the effects of different types of leisure activities on cognitive function in older adults.

The study findings would help optimize future interventions to promote normal cognitive aging in older adults.

## 2. Materials and methods

This study was conducted and reported according to the Preferred Reporting Items for Systemic Review and Meta-Analysis Statement (PRISMA) 2020 guidelines (Page et al., 2021). This systematic review and meta-analysis was pre-registered in the International Prospective Register of Systematic Reviews (registration number: CRD42022301199).

### 2.1. Search methods

We identified relevant studies published from January 2012 to January 2022 by searching MEDLINE, PubMed, EMBASE, PsycINFO (Ovid), CINAHL (EBSCO), and Web of Science. Search strings included suitable indexing terms (e.g., MeSH terms and keywords) on “leisure activities” AND “cognition” AND “aged” AND “longitudinal” (Supplementary Section 1). After removing duplicates, two reviewers screened all titles and abstracts independently. The reviewers then independently assessed the full articles according to the inclusion/exclusion criteria. Any disagreement was resolved by consensus or by consulting with a third reviewer.

### 2.2. Search criteria

We included studies with the following criteria:

1. Published in English
2. The full text was available
3. Participants were 65 years or older, and free of cognitive impairment at baseline
4. All or some of the outcome indicators included were cognitive function, which was assessed using neuropsychological tests
5. The effect of leisure activities (e.g., physical, cognitive, and social activities) on the cognitive function was reported
6. Observational longitudinal studies with a follow-up of least 1 year.

We excluded studies with the following criteria:

1. Qualitative studies, case studies, reviews, interventional studies, or conference papers
2. Participants with other illnesses that affect cognitive function (e.g., Parkinson’s disease, epilepsy, amyotrophic lateral sclerosis, Huntington’s Disease, Schizophrenia, brain damage, and vascular cognitive impairment)
3. Insufficient data to calculate the relative risks (RRs).

### 2.3. Data extraction

Two reviewers extracted the following data: country, study design, data source, length of follow-up, inclusion and exclusion criteria, sample size, mean age, percentage of females, assessments and types of leisure activities, and cognitive function. Crude and adjusted RRs representing the effect of leisure activities on cognitive decline, cognitive impairment, and dementia in older adults were extracted. Estimates adjusted for potential confounders were used for the meta-analyses where possible. Inconsistencies were resolved by consensus with the third reviewer through discussion between the two reviewers.

### 2.4. Quality assessment

Two reviewers independently assessed the risk of bias of each study according to the Scottish Intercollegiate Guidelines Network checklists developed by researchers in Scotland (Sun et al., 2013). Any disagreement was resolved by consensus meetings.

### 2.5. Data synthesis and analysis

The RR and related 95% CI were calculated if a study provided raw data without RR. If the hazard ratios (HRs) or odds ratios (ORs) were reported for a study, we calculated the RR by using the HR or OR reported in the original study and the control event rate (*P_0_*; Supplementary Section 2; Shor et al., 2017; Jike et al., 2018). For studies that reported neither the RR nor *P_0_*, the P0 was obtained from studies with similar characteristics (Shor et al., 2017; Jike et al., 2018). The regression coefficient were converted to logOR and subsequently to OR, which was used to calculated the RR (Shor et al., 2017).

All statistical analyses were performed by Stata, version 17.0. First, RRs were combined through the fixed effect model. If the heterogeneity test was statistically significant, the random-effect model was then applied. Subgroup analyses were performed according to the type of leisure activities, and different cognitive statuses. Based on the leisure activity classification of the included studies, we divided leisure activities into three categories: physical (e.g., light or brisk walking, calisthenics, gateball, golf, dancing, jogging, hiking, bowling, cycling, swimming, Tai Chi, or yoga, etc.), cognitive (e.g., reading books, newspapers, or magazines, watching television or listening to the radio, etc.), and social activities (e.g., attending religious activities, engagement in social work, traveling, etc.). If the included studies did not classify activities, we classified activities based on the procedure used in other included studies and previous studies (Verghese et al., 2006; Wang et al., 2012; Zhang et al., 2021a). The cognitive status was divided into three categories: dementia, cognitive impairment, and cognitive decline. In this study, cognitive impairment referred to cognitive impairment without dementia, which included all individuals with cognitive impairment whose severity was insufficient to meet the diagnostic criteria for dementia (Graham et al., 1997). Cognitive decline was defined as a decline in participants’ scores on measures of cognitive function from baseline to follow-up (Lee A. T. C. et al., 2015; Osuka et al., 2020; Endeshaw and Goldstein, 2021). Specific diagnostic criteria for dementia, cognitive impairment and cognitive decline were based on the criteria in the included studies.

Heterogeneity between studies was tested using the Cochran’s Q statistic (*p* < 0.05 was considered statistically significant) and *I*^2^ statistic (*I^2^* > 50% was considered to indicate substantial heterogeneity; Cumpston et al., 2019). Meta-regression was used to investigate potential sources of heterogeneity between studies (Cumpston et al., 2019; Page et al., 2021). We conducted a univariate meta-regression for the following pre-specified characteristics: mean age of participants, type of cognitive outcome (dementia, cognitive impairment, and cognitive decline), type of leisure activities (physical, cognitive, and social activities), percentage of female participants (≥50%, and <50%), sample size, country (European and American countries, and Asian countries), follow-up (≤3 years, and >3 years), cognitive assessment intervals (≤2 years, and >2 years), number of cognitive assessments, and cognitive assessment measures (the Mini Mental State Examination (MMSE), MMSE and other measures, and other measures). The effect of each exploratory variable on RR was obtained using exponentiated coefficients (*exp(β)*). For *exp(β)* > 1, the percentage increase in RR is calculated as (*exp(β)*− 1) × 100%. For *exp(β)* < 1, the percentage decrease in RR was calculated as (1 − *exp(β)*) × 100%. Furthermore, variables with *p* < 0.20 in univariate analysis were included in the multivariate regression model (Maldonado and Greenland, 1993). Sensitivity analysis was used to assess the stability of the results by excluding one study at a time to identify the effect of any individual study on the pooled effect size and between-study heterogeneity. Publication bias was assessed by producing a contour-enhanced funnel plot (Lassale et al., 2019), and the trim and fill method was also applied (Mavridis and Salanti, 2014). We used Egger’s method to test the asymmetry of the funnel plot, and publication bias was assumed with *p* < 0.10.

## 3. Results

### 3.1. Study selection

**Figure 1:**
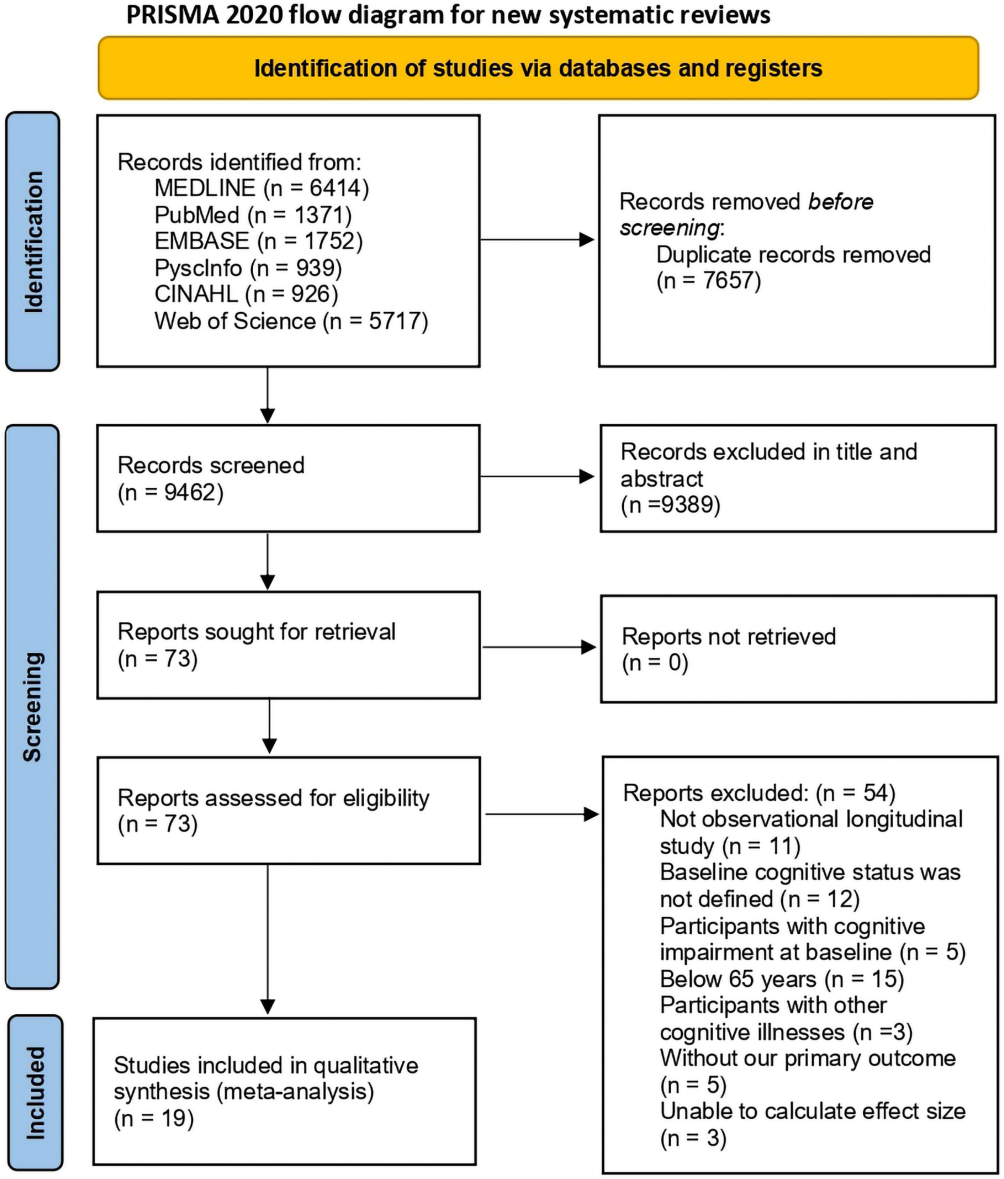
Flow chart of the selection process for this meta-analysis.

We initially identified 17,119 studies from database search. Among them, 7,657 duplicates were excluded. After excluding abstracts, conference papers, interventional studies, cross-sectional studies, and animal studies, 73 studies were selected for full-text review. After reviewing the full-texts, 54 studies were excluded, among which: 11were not observational longitudinal studies, 12 did not specify the baseline cognitive status of participants, 5 included participants with cognitive impairment at baseline, 15 included participants below the age of 65 years, 3 included participants with other cognitive illnesses (e.g., Parkinson’s disease), 5 did not contain information about our primary outcome (the effect of leisure activities on cognitive function), and 3 did not calculate the effect size. Resultantly, 19 studies were included in meta-analyses of this study. Figure 1 shows the PRISMA flow chart depicting the study selection process.

### 3.2. Study characteristics

The characteristics of included studies are presented in Table 1, and more information is presented in Supplementary Table S1. All of the included studies were prospective cohort studies. Among them, 8 studies were conducted in European and American countries, whereas 11 studies were conducted in Asia. The mean age of participants ranged from 71.4 to 89.2 years with a follow-up duration ranging between 1.0 and 16.0 years. The number of participants ranged from 687 to 73,260. The percentage of female participants varied between studies, and one study included only women (Osuka et al., 2020). According to quality appraisal, all studies were rated as “++.”

**Table 1:** Characteristics of the included studies.

Author (Year)	Country	Follow-up*	Sample size	Mean age (SD), range (years)	Female (%)	Activity type	Activity assessments	Outcome	Cognitive assessments	Study quality
Dupré et al. (2020)	Canada	2.0	1,271	77.6, 65+	50.8	Physical activity	The self-report Voorrips questionnaire: All leisure time and sport activities were pooled in the leisure/sport activity sub-score (intensity* number of hours per week* number of months per year).	Dementia	Global cognition: MoCA	++
Dupré et al. (2021)	France	8.0	1,697	79.9, 65+	36.5	Physical activity	The Physical Activity Scale for the Elderly: the frequency, duration and intensity level of activities	Dementia	Global cognition: MMSE Visual working memory: BVRT Psychomotor speed and executive functions: TMTA and TMTB Verbal fluency: the Isaacs’s Set Test Verbal episodic memory: FCSRT	++
Endeshaw and Goldstein (2021)	America	Range: 1.0–3.0	4,227	75.7 (7.1), 65–102	58.0	Physical activity	Self-reported information	Cognitive decline	Memory: immediate and delayed free recall of 10 words Executive function and Visuo-spatial ability: CDT Orientation: orientation to time and current events	++
Fajersztajn et al. (2021)	Brazil	2.0	1,243	71.7 (5.8), 65+	61.4	Cognitive activity	The Brazilian version of the ‘Involvement in Activities’ questionnaire	Dementia	Global cognition: COGSCORE Immediate memory: a list of 10 words adapted from the CERAD Verbal fluency: animal naming	++
Hughes et al. (2015)	America	1.8	864	78.3 (6.8), 65+	63.3	Cognitive activity; Physical activity	The Florida Cognitive Activities Scale and self-reported information	Cognitive impairment	Global cognition: CDR	++
Kishimoto et al. (2016)	Japan	11.5 (median)	803	74.0, 65+	61.0	Physical activity	A self-administered questionnaire on life-style: the frequency of such activity per week and the time spent in each session during the past month	Dementia	Global cognition: HDS, HDS-R, or MMSE	++
Krell-Roesch et al. (2019)	America	5.0 (median)	2000	78.3, 70+	50.1	Cognitive activity	A structured survey with ordinal responses	Cognitive impairment	Global cognition: CDR Memory: AVLT-H, WMS-R, LM Language: BNT and CRF Visuospatial skills: WAIS-R, Picture Completion, and Block Design subtests Attention/executive function: TMTB, DSST, and WAIS-R	++
Lee et al. (2018)	China	5.0 (median)	15,589	74.0 65+	63.9	Cognitive activity	Nurses used a questionnaire to ascertain the frequency and type of leisure activities that the participants practiced in the prior month	Dementia	Global cognition: MMSE and CDR Memory: a 3-object delayed recall test:	++
Lee A. T. C. et al. (2015)	China	6.0	2,605	74.2, 65+	63.9	Physical activity	The Elderly Health Centers nursing staff to describe the duration, frequency, and type of habitual physical exercise that they practiced in the past 1 month	Cognitive decline	Global cognition: MMSE	++
Lee Y. et al. (2015)	Korea	2.0	15,582	71.9 (6.6), 65+	55.5	Physical activity	Self-reported information: the type, frequency, and duration	Dementia	Global cognition: DWR, AMT, MMSE, CDR	++
Mao et al. (2020)	China	3.4 (median)	10,741	88.0, 80+	54.4	Cognitive activity; Social activity; Physical activity	Self-reported information: the type, frequency	Cognitive impairment	Global cognition: MMSE	++
Ogino et al. (2019)	America	4.1	1,345	75.0 (6.3), 65+	68.0	Physical activity	The Godin leisure time exercise questionnaire: the frequency of leisure time physical activity during the most recent 2-week period, and duration (minutes) per session	Cognitive impairment	the neuropsychological test	++
Osuka et al. (2020)	Japan	Range: 1.0–2.0	687	71.4, 65–81	100.0	Physical activity	A self-developed questionnaire: overall exercise duration, frequency per week, and length of the continuous period for each exercise type	Cognitive decline	MMSE	++
Qiu et al. (2019)	China	16.0 (median)	4,830	89.2, 80+	48.1	Cognitive activity	The questionnaire at baseline: the frequency	Cognitive impairment	Global cognition: MMSE	++
Sato et al. (2021)	Japan	5.7	73,260	73.9, 65+	53.5	Physical activity	Self-reported information: the frequency of physical activity per week	Dementia	Global cognition: MMES; CDR	++
Yoon et al. (2021)	Korea	42 months (median)	62,286	73.2, 65+	60.4	Physical activity	Self-report–structured questionnaires: the usual frequency (days per week)	Dementia	Global cognition: KDSQ	++
Zhang et al. (2021a)	China	6.0	3,017	77.0 (9.0), 65+	49.3	Cognitive activity; Social activity; Physical activity	Self-reported information: the frequency	Cognitive decline	Global cognition: MMSE	++
Zhou et al. (2017)	China	9.0	7,501	82.1, 65+	54.6	Physical activity	Self-reported information	Dementia	self- or proxy-report of a doctor’s diagnosis	++
Zhu et al. (2017)	China	4.6	6,586	79.5 (9.8), 65–105	51.7	Cognitive activity; Physical activity	Self-reported information: the frequency	Cognitive impairment	Global cognition: MMSE	++

^*^Fllow-up: mean years, unless otherwise specified. MMSE, The Mini Mental State Examination; TMTA, The Trail Making Test Part A; TMTB, The Trail Making Test Part B; HVLT-R, The Hopkins Verbal Learning Test; MoCA, The Montreal Cognitive Assessment; BVRT, The Benton Visual Retention Test; IST, The Isaacs’s Set Test; FCSRT, The Free and Cued Selective Reminding Test; Word Recall, immediate and delayed free recall of 10 words; CDT, clock drawing test; COGSCORE, the cognitive score validated by the 10/66 Dementia Research Group; CERAD, the Consortium to Establish a Registry for Alzheimer’s Disease battery; DSM-IV, the Diagnostic and Statistical Manual of Mental Disorders, 4th edition; CDR, The Clinical Dementia Rating scale; HDS, the Hasegawa’s Dementia Scale; HDS-R, the Hasegawa Dementia Scale-Revised; AVLT-H, delayed recall trials from Auditory Verbal Learning Test; WMS-R, Wechsler Memory Scale–Revised; LM, Logical Memory; VR, visual reproduction subtests; BNT, Boston Naming Test; CRF, category fluency; WAIS-R, Wechsler Adult Intelligence Scale–Revised; DSST, Digit Symbol Substitution subtest; DWR, the Delayed Recall Test; AMT, the Abbreviated Mental Test; KDSQ, the Korean Dementia Screening Questionnaire.

### 3.3. Meta-analysis

**Figure 2:**
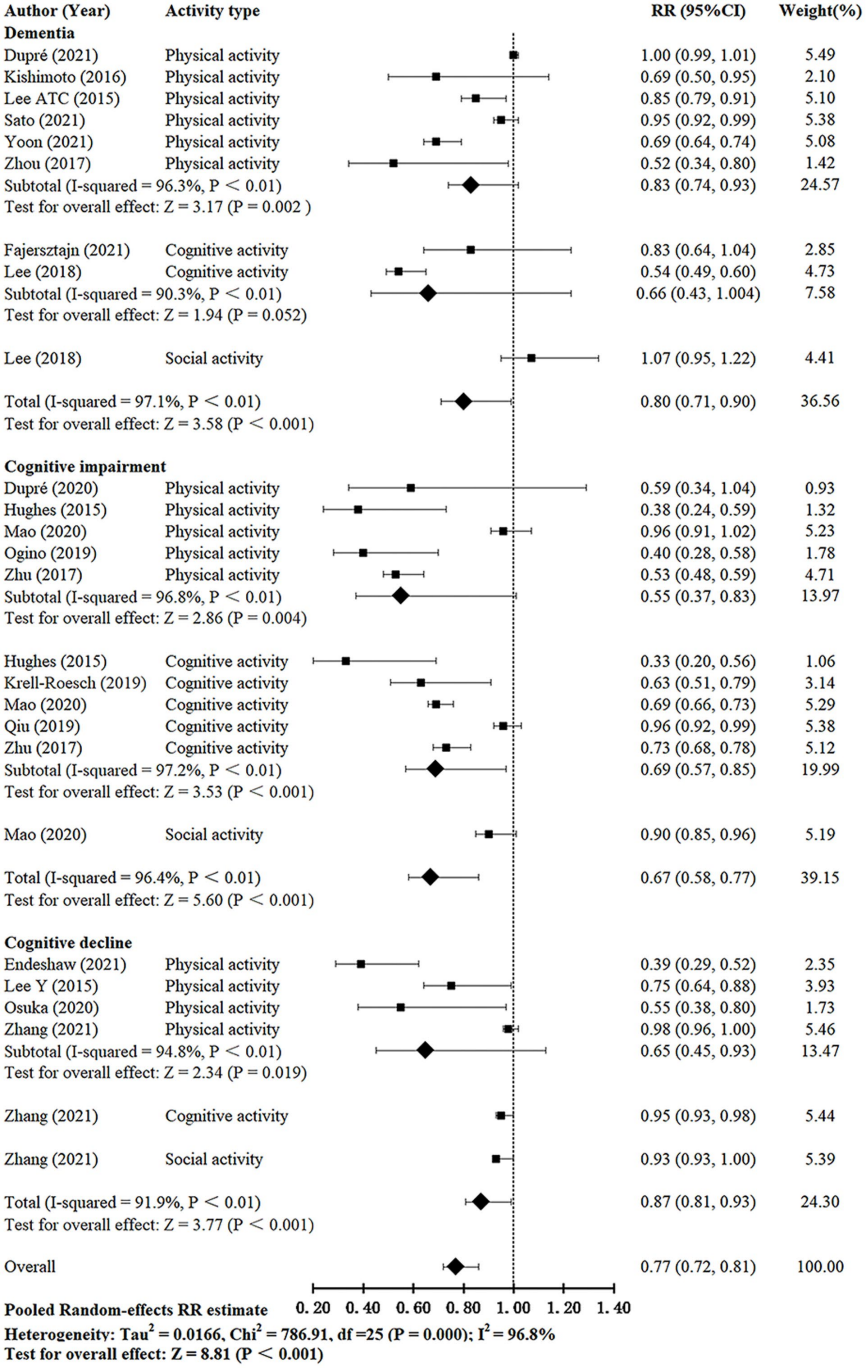
Meta-analysis of the relationship between leisure activity and cognitive function in older adults.

There was considerable heterogeneity between studies (*I^2^* = 96.8%, Q-test: *p* < 0.01). The effects of leisure activities on the cognitive outcomes of interest were summarized, and the forest plot is shown in Figure 2. The pooled RR for the effect of leisure activities on cognitive function was 0.77 (95% CI, 0.72–0.81, *p* < 0.001).

#### 3.3.1. Effect of leisure activities on dementia risk

Eight studies (Lee A. T. C. et al., 2015, 2018; Kishimoto et al., 2016; Zhou et al., 2017; Dupré et al., 2021; Fajersztajn et al., 2021; Sato et al., 2021; Yoon et al., 2021) investigated the effect of leisure activities on dementia in older adults. The *I*^2^ was 97.1%. The pooled RR for leisure activities on dementia was 0.80 (95% CI: 0.71–0.90, *p* < 0.001). The pooled RRs for each subgroup (except social activity) were calculated; physical and cognitive activities showed pooled RRs of 0.83 (95% CI: 0.74–0.93, *p* = 0.002) and 0.66 (95% CI, 0.43–1.004, *p* = 0.052), respectively. Studies that evaluated cognitive activity reported lower pooled RRs compared with those of activities.

#### 3.3.2. Effect of leisure activities on cognitive impairment risk

Seven studies (Hughes et al., 2015; Zhu et al., 2017; Krell-Roesch et al., 2019; Ogino et al., 2019; Qiu et al., 2019; Dupré et al., 2020; Mao et al., 2020) investigated the effect of leisure activities on cognitive impairment in the older population. The *I^2^* was 96.4%. The pooled RR for leisure activities on cognitive impairment was 0.67 (95% CI: 0.58–0.77, *p* < 0.001). The pooled RRs for each subgroup (except social activity) were calculated; physical and cognitive activities showed pooled RRs of 0.55 (95% CI: 0.37–0.83, *p* = 0.004) and 0.69 (95% CI: 0.57–0.85, *p* < 0.001). Studies that evaluated physical activity reported lower pooled RR compared with those of other activities.

#### 3.3.3. Effect of leisure activities on cognitive decline risk

Four studies (Lee Y. et al., 2015; Osuka et al., 2020; Endeshaw and Goldstein, 2021; Zhang et al., 2021a) investigated the effect of leisure activities on cognitive decline in the older population. The *I^2^* was 91.9%. The pooled RR for leisure activities on cognitive decline was 0.87 (95% CI: 0.81–0.93, *p* < 0.001). The pooled RR for the physical activity group was 0.65 (95% CI: 0.45–0.93, *p* = 0.019).

### 3.4. Meta-regression

Univariate meta-regression analysis was performed to investigate the reason of the relatively high heterogeneity among studies (Table 2). Follow-up, cognitive assessment intervals, cognitive assessment measures, country, percentage of female participants, and type of leisure activity were associated with logRR. Studies conducted in European and American countries had significantly lower RRs (*exp(β)* = 0.718, 95% CI: 0.548–0.941, *p* = 0.019) than studies conducted in Asian countries. Compared with studies with female participant percentage ≥50%, those with female participant percentage <50% had significantly increased RRs (*exp(β)* = 1.445, 95% CI: 1.100–1.898, *p* = 0.010). Follow-up, cognitive assessment intervals, cognitive assessment measures, country, and percentage of female participants can explain the heterogeneity.

**Table 2:** Meta-regression of exploring factors contributing to heterogeneity in the relative risk.

Moderator	*exp(β)*	95% CI	*p*-value	*R* ^2^
**Mean age**	1.011	(0.869,1.036)	0.383	0.029
**Simple size**	1.000	(1, 1)	0.347	0.011
**Number of cognitive assessments**	1.001	(0.893, 1.121)	0.923	0.044
**Follow-up**			0.015**	0.241
≤3 years	1			
>3 years	1.439	(1.081, 1.917)		
**Cognitive assessment intervals**			0.087*	0.141
>2 years	1			
≤2 years	0.788	(0.598, 1.039)		
**Cognitive assessment measures**			0.004**	0.402
MMSE and other measures	1			
MMSE	1.059	(0.807, 1.392)	0.666	
Other measures	0.677	(0.498, 0.922)	0.015**	
**Country**			0.019**	0.189
Asian country	1			
The European and American country	0.718	(0.548, 0.941)		
**Female percentage**			0.010**	0.299
≥50%	1			
<50%	1.445	(1.100, 1.898)		
**Type of leisure activity**			0.202	0.088
Social activity	1			
Physical activity	0.705	(0.476, 1.044)	0.079*	
Cognitive activity	0.731	(0.481, 1.113)	0.139	
**Type of cognitive outcome**			0.397	0.052
Cognitive decline	1			
Cognitive impairment	0.864	(0.615, 1.214)	0.384	
Dementia	1.051	(0.741, 1.492)	0.770	
**Multivariable *R*** ^**2**^ **of the model**			0.010**	0.665

MMSE, the Mini Mental State Examination. **p < 0.05, *p < 0.20.

### 3.5. Publication bias

**Figure 3:**
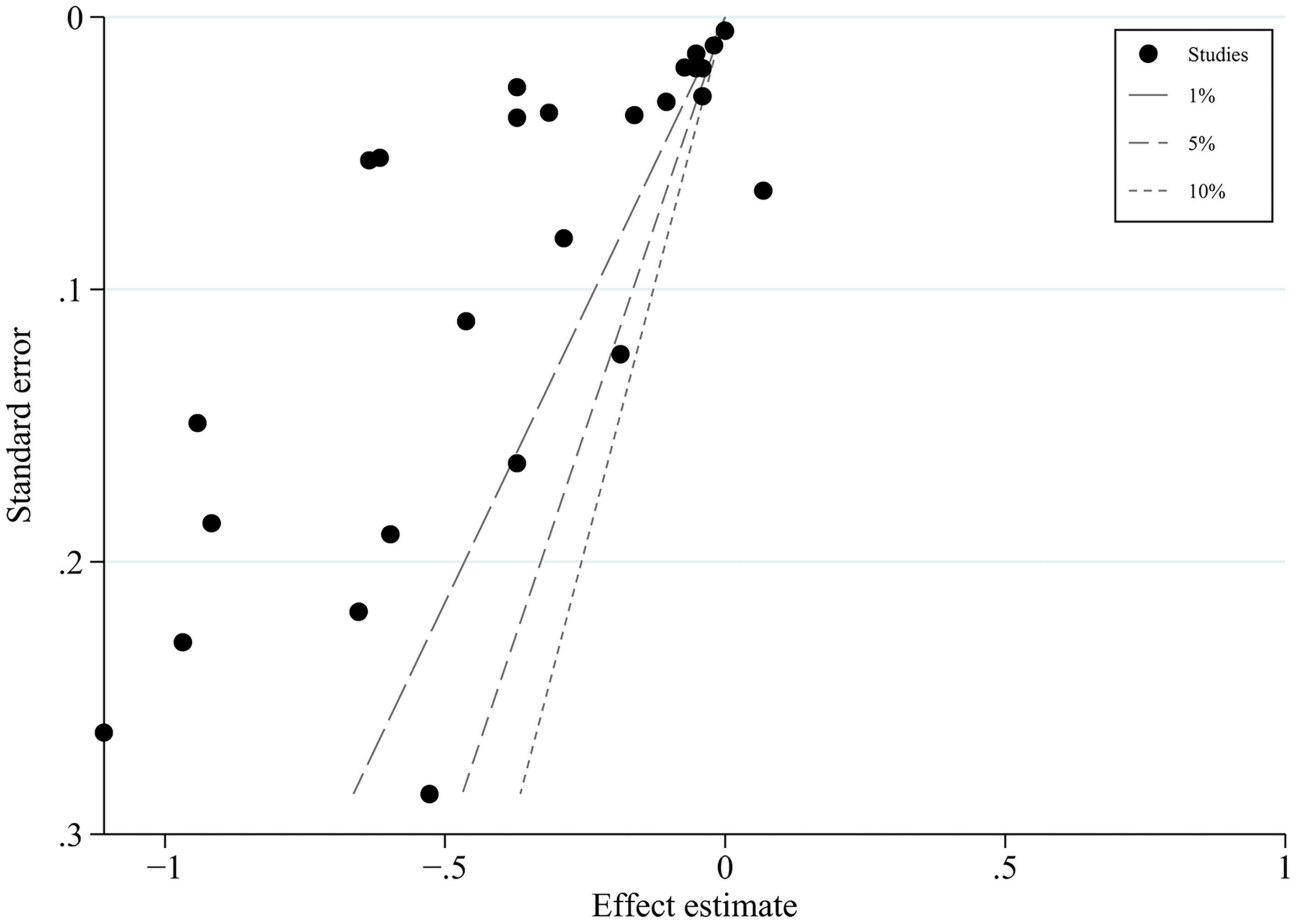
One-sided contour-enhanced funnel plot.

Egger’s test (*p* < 0.01) and the contour-enhanced funnel plot showed the risk of publication bias (Figure 3). In contrast, the trim-and-fill method for publication bias showed that it was not necessary to trim any existing study and fill any additional unpublished study. Therefore, this study was considered to have no significant risk of publication bias, but there were bias due to other factors (Lee et al., 2019).

### 3.6. Sensitivity analysis

**Figure 4:**
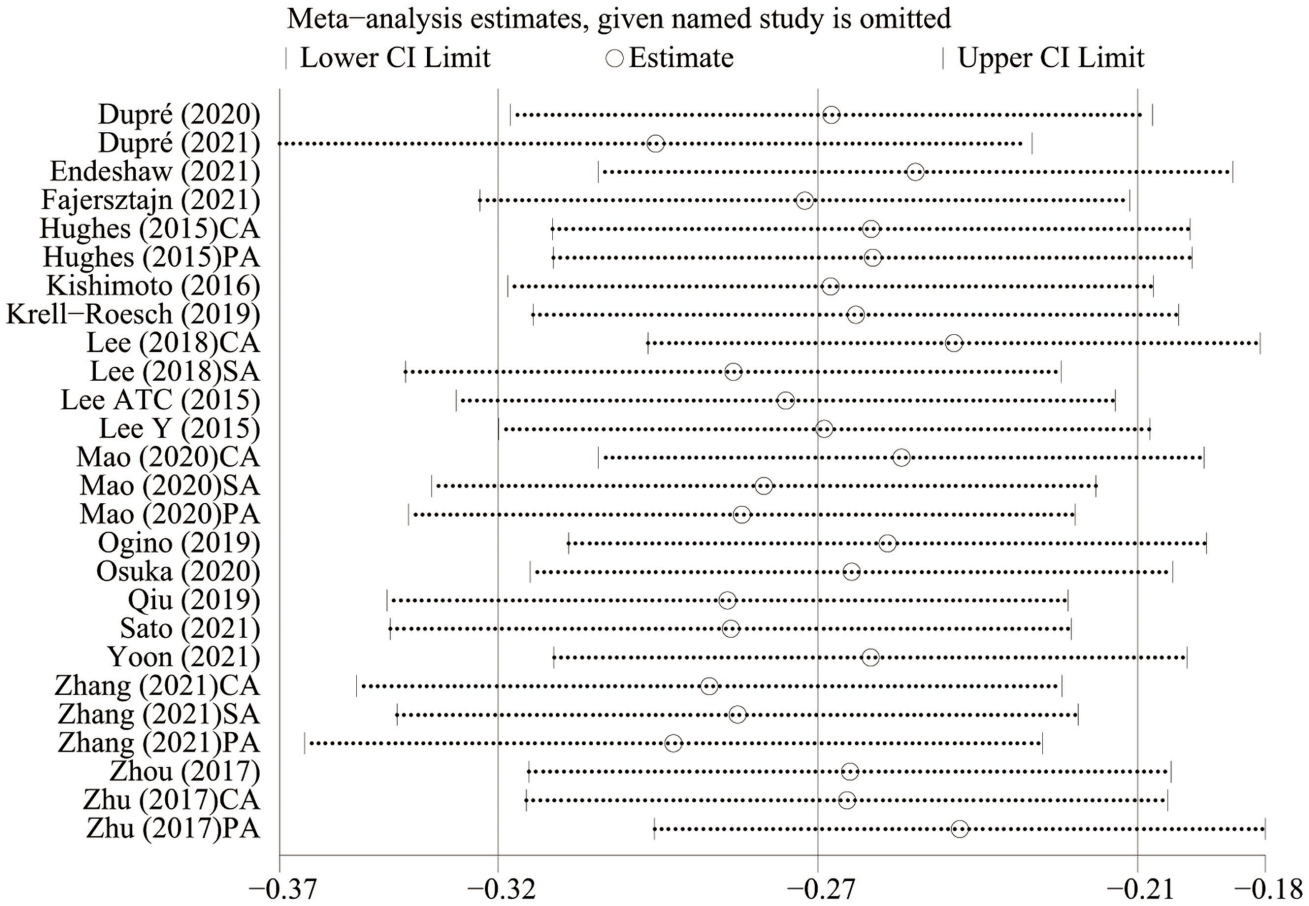
Plot of sensitivity analysis by excluding one study each time and the pooling estimate for the remaining studies. CA, cognitive activity; SA, social activity; PA, physical activity.

We performed sensitivity analysis by excluding one study at a time to identify the effect of any individual study on the pooled effect size and between-study heterogeneity. No study significantly affected the pooled effect size (Figure 4).

## 4. Discussion

Our study highlighted the positive effect of leisure activities on the protection of cognitive function in older adults. The result was consistent with those of previous studies (Wang et al., 2012; Sajeev et al., 2016; Wollesen et al., 2020). Previous reviews have explored and compared the effects of various dietary patterns and multiple lifestyles on cognitive aging (Dominguez et al., 2021). However, comparisons regarding the effects of various leisure activities on cognitive aging are still lacking. This systematic review and meta-analysis showed that different types of leisure activities posed different effects on different cognitive statuses.

We identified that leisure activities had a positive effect on all three different cognitive statuses in older adults. In the subgroup analysis, the effect of leisure activities was more prominent in delaying onset of cognitive impairment. Older adults at different cognitive statuses may have various substantial changes in brain structures (Leong et al., 2017). A study found that a small number of people with cognitive impairment can finally return to cognitive normalcy (Zhang et al., 2021b). However, some scholars have suggested that some brain structural changes are irreversible in people with symptoms of dementia (Kim et al., 2019). With the new understanding of the effects of leisure activities on different cognitive statuses in older adults, future studies are required to explore more evidence on the existence of these effects.

We also performed a subgroup meta-analysis of the effect of different types of leisure activities on cognitive function in older adults. Our study showed that cognitive activity did not have a significant effect on the delay in the onset of dementia (RR: 0.66, 95% CI: 0.43, 1.004, *p* = 0.052). However, the findings of some reviews indicated that cognitive activity had a positive effect on the delay in the onset of dementia (Wang et al., 2012; Sajeev et al., 2016). The inconsistency between our findings and the abovementioned previous study findings may be explained by the small number of studies included in the subgroup analysis. Moreover, the inconsistency may be related to differences in the types of cognitive activities evaluated in the included studies. Some included studies showed that watching television positively affect cognitive function in older adults. Lee et al. and Shin et al. also had the similar findings (Lee et al., 2018; Shin et al., 2021). In addition, many studies have found that other cognitive activities like reading books, using a computer, and playing cards/games/solving puzzles had positive effects on the delay in the onset of dementia (Shin et al., 2021). Therefore, more studies are need to clarify and explore the effects of different types of cognitive activities on the delay in the onset of dementia.

We also identified that social activities did not significantly affect the delay in the onset of cognitive decline in older adults. However, Pugh et al. reported that social activity had a positive effect in reducing the occurrence of cognitive decline (Pugh et al., 2021). The inconsistency in study fundings may be related to differences in the types of social activities included in the studies. Kim et al. found that personal social activities (meeting with close friends) did not significantly delay cognitive decline in older adults (Kim et al., 2017). Nevertheless, two social group activities (social club/café and alumni) significantly delayed cognitive decline (Kim et al., 2017). Health care recommendations aimed at delaying cognitive decline in older adults should target the promotion of their participation in social group activities.

Substantial heterogeneity was found in this study. After examining this heterogeneity carefully by meta-regression analysis, we found that the most possible underlying causes were certain methodological differences in follow-up, cognitive assessment intervals, cognitive assessment measures, country, and percentage of female participants. Sensitivity analyses showed that no study significantly affected the pooled effect size. Therefore, the results of this meta-analysis are reliable and stable. Meta-regression showed that the studies with female participant percentage <50% reported a larger protective effect of leisure activities on cognitive function in older adults than studies with female participant percentage ≥50%. This may be due to gender differences in the effect of leisure activities on cognitive function in older adults. A recent study showed that although men and woman had the same level of participation in total leisure activities, men were more engaged in self-improvement activities than women (Hassing, 2020). Therefore, leisure activities may elicit a greater effect on the cognitive function in men than in women. Gender differences should be considered when examining the effect of leisure activities on cognitive function in older adults. In addition, gender differences should be considered in the development of interventions to preserve cognitive function in older adults.

This meta-regression analysis also found that studies conducted in European and American countries had significantly lower RR than studies conducted in Asian countries. This may be related to the different types and frequencies of older adult participation in leisure activities in different countries. The sociodemographic characteristics, socioeconomic status (including education level, income level, and employment status), and health status of older persons vary across countries (Minicuci et al., 2019). These factors may also influence the effect of leisure activities on cognitive function in older adults. However, there is currently a lack of studies comparing the effects of leisure activities on cognitive function in older adults in different countries. Future studies should further explore the substantial differences and reasons for the differences in the effects of leisure activities on the cognitive function of the elderly across countries. Identifying differences may lead to improved health care recommendations and interventions across countries to ameliorate cognitive function in older adults and promote healthy cognitive aging globally.

In addition, previous studies have reported that factors including frequency and intensity of activity, specific type of activity, age of engagement in activity, gender, and education level may influence the effect of leisure activities on cognitive function in older adults (Krell-Roesch et al., 2019; Bielak and Gow, 2022). The effect of leisure activities on cognitive function in older adults is more pronounced for the low education level than for the high education level (Ihle et al., 2015), but the evidence is inconsistent (Kishimoto et al., 2016; Stenling et al., 2021). Few studies have investigated the moderating role of education in the relationship between activity and cognitive function. More studies are needed to explore the role of education in the relationship between activity and cognitive function, and the associated mechanisms. Furthermore, a study found that only doing craft activities in later life had a positive effect on the cognitive function in older adults, and only performing reading activities in both middle and late life had a positive effect on the cognitive function in older adults (Krell-Roesch et al., 2019). Future studies should consider the influence of the above factors on the effect of leisure activities on cognitive function in older adults, and therefore to provide an evidence base for developing and strengthening targeted intervention programs.

Our study has several strengths. First, all included studies were prospective cohort studies. All case–control or cross-sectional studies were excluded to minimize recall bias. Furthermore, meta-regression analysis was performed to investigate the effect of different countries on the variation in RRs. Moreover, our study examined the effects of different types of leisure activities on different cognitive statuses, which was not performed in previous reviews (Verghese et al., 2006; Wang et al., 2012). In this meta-analysis, we investigated the effect of different types of leisure activities on different cognitive statuses; our findings can provide a direction for future study to explore the optimal type of leisure activities for intervention in older adults with different cognitive statuses.

Although our meta-analysis found protective effects of leisure activities on cognitive function in older adults, some study limitations that should be considered. First, there was a high degree of heterogeneity among the included studies. Although our meta-estimates were derived from cohort studies, which may exhibit a high degree of heterogeneity, analyses of such studies provided results that can be considered similar to those of randomized trials (Anglemyer et al., 2014). Second, in most of the included studies, leisure activities were self-reported, which may lack objectivity and accuracy. Furthermore, we grouped activities according to the primary classification of retrieved studies, and could not differentiate the components of the activity. Future studies should consider differentiating the components of the activity to avoid neglecting the role of the non-dominant part of the activity, and identify constellations of (social, physical, cognitive) activities that are particularly beneficial about cognitive aging. Third, participation in activities may have different various benefits for cognitive subdomains in older adults. Unfortunately, this meta-analysis cannot distinguish the effects of various types of activities on cognitive subdomains. Futher studies should clarify and quantify the effects of different frequencies and intensities of activities on different cognitive subdomains in older adults.

In conclusion, the present systematic review and meta-analysis provided evidence that multiple types of leisure activities, especially physical activities had positive effects on cognitive function in older adults. Therefore, we recommend that older adults should perform more leisure activities to promote their cognitive function. Moreover, different types of leisure activities were found to elicit different effects on cognitive function in older adults. However, more data are needed to confirm these effects. Future studies should investigate the optimal type, duration, intensity, regional and gender difference of leisure activities, and the optimal age of engagement in leisure activities that preserve cognitive function in older adults.

## Author contributions

XY, XX, LG, YZ, and YL contributed to the study conception and design. Study screening, data extraction, and assessment of study quality were performed by XY, XX, LG, and YZ. Validation and data curation were performed by XY and XX. The first draft of the manuscript was written by XY, XX, and SW. All authors contributed to the article and approved the submitted version.

## Funding

This study was supported by the National Natural Science Foundation of China (grant nos. 72204075 and 72204228), the Hebei Provincial Postdoctoral Science Foundation (grant no. B2022003032), and the Science and Technology, Scientific Research Foundation Program of School of Nursing, Hebei Medical University (H2020003).

## Conflict of interest

The authors declare that the research was conducted in the absence of any commercial or financial relationships that could be construed as a potential conflict of interest.

## Publisher’s note

All claims expressed in this article are solely those of the authors and do not necessarily represent those of their affiliated organizations, or those of the publisher, the editors and the reviewers. Any product that may be evaluated in this article, or claim that may be made by its manufacturer, is not guaranteed or endorsed by the publisher.

## Supplementary material

The Supplementary material for this article can be found online at: https://www.frontiersin.org/articles/10.3389/fpsyg.2022.1080740/full#supplementary-material
