# Supplementary material for: Effect of leisure activities on cognitive aging in older adults: A systematic review and meta-analysis
Source: Front Psychol. 2022 Dec 22;13:1080740. doi: 10.3389/fpsyg.2022.1080740 (PMC9815615; doi:10.3389/fpsyg.2022.1080740)
Supplement: Supplementary file 1 [file Table_1.DOCX]

Supplementary Material

# Section 1: Search strategies and search terms

“Cognition” AND “Elderly” AND “Exposure” AND “Study design”

| Cognition | **“cognition”** OR **“neuropsychological tests”** OR **“cognitive function”** OR “cognitive ability” OR “executive function” OR “working memory” OR **“dementia”** OR **“cognitive dysfunction”** OR **“Alzheimer's disease”** OR “cognitive impairment” OR “cognitive decline” |
| --- | --- |
| Elderly | **“aged”** OR **“aging”** OR “elderly” OR “older” OR “old adults” OR “old people” |
| Exposure | **“leisure activities”** OR “productive activity” OR “physical activity” OR **“exercise”** OR **“sports”** OR “aerobic exercise” OR “mind–body exercise” OR “cognitive activity” OR “mental activity” OR “intellectual activity” OR “cognitive stimulation” OR “cognitive leisure activity” OR “cognitively-stimulating activities” OR “social activity” OR “social engagement” |
| Study design | **“longitudinal”** OR **“cohort”** OR **“prospective”** |

Terms in bold are taken from MeSH

# Section 2: Additional information on the conversion of odds ratios and hazard ratios to rate ratios

If hazard ratios or odds ratios were reported for a study, we calculated rate ratios by using the hazard ratios or odds ratios reported in the original study and the control event rate. The equations used are as follows:


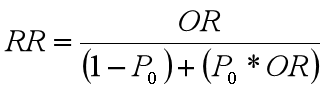


or


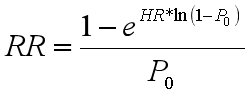


where RR is the relative risk, OR is the odds ratio, HR is the hazard ratio, and P0 is the control event rate. For studies that reported neither RR nor P0, P0 was borrowed from studies with similar characteristics.

# Table S1. Characteristics of the included studies

| **Author (Year)** | **In- and ex-clusion criteria** | **Covariates** | **Number of cognitive assessment** | **Cognitive assessment intervals (years)** |
| --- | --- | --- | --- | --- |
| Dupré (2020) | Inclusion: living in a private household and not being hospitalized Exclusion: had cognitive disorders at the baseline | age, gender, education, depression, arthritis, stroke, diabetes, frailty | 5 | 0.5 |
| Dupré (2021) | Inclusion: aged 65 years or older without dementia at baseline | age, gender, study center, education, depression, alcohol, BMI, benzodiazepine, diabetes, hypertension, APOE 4 allele, consumption of fruits and vegetables, cardiovascular disease, and time by age, study center, depression, BMI, diabetes, hypertension and APOE4 allele interactions | 4 | 2 |
| Endeshaw (2021) | Exclusion: (a) live in residential facilities (b) were told to have dementia or Alzheimer’s disease by their health care provider at baseline (c) participants whose cognition test showed impairment in one or more cognitive domains at baseline (d) participants who did not complete cognitive function test during at least one follow-up period | age, gender, marital status, race/ethnicity, education, chronic diseases, social activity and depression symptoms | 4 | 1 |
| Fajersztajn (2021) | Inclusion:  aged 65 years or over, with no dementia diagnosis at baseline | the corresponding baseline score, age, gender, marital status, schooling, occupation, personal income and function status | 2 | 2 |
| Hughes (2015) | Inclusion: (a) community-dwelling elders (b) aged 65 years or older  Exclusion: based on an age-education adjusted MMSE score < 21/30 | age, gender, race, education, general mental status, depressive symptoms, central obesity (waist-to-hip ratio, continuous), subjective rating of health, and number of prescription medications dichotomized at the sample median value of 4 | 2-4 | 1 |
| Kishimoto (2016) | Inclusion: (a) community-dwelling elderly Japanese individuals  (b) without dementia (c) aged ≥65 years | age, gender, education, systolic blood pressure, antihypertensive agents, diabetes, total cholesterol, BMI, electrocardiogram abnormalities, history of stroke at entry, smoking habits, and alcohol consumption | 3 | 6.5 |
| Krell-Roesch (2019) | Inclusion: (a) cognitively unimpaired individuals (b) aged ≥70 years  (c) had completed a self-reported questionnaire on engagement in midlife and late-life mentally stimulating activities at baseline (d) had undergone cognitive evaluation at baseline and on average every 15 months during follow-up | age, gender, education, and APOE geno-type status | 2-8 | 1.25 |
| Lee (2018) | Inclusion: (a) aged 65 years or older (b) Chinese ethnicity (c)living in the community Exclusion: (a) non- Chinese ethnicity (b) living in care homes (c) having history of stroke, Parkinson disease, or clinical dementia (d) scoring below the education-specific cutoff on the C-MMSE at baseline | age, gender, educational, cardiovascular risk factors, visual and hearing impairments, poormobility, depression,smoking, adequate fruit andvegetable intake, regularphysical exercise, and other types of leisure activities | ≥2 | ≥3 |
| Lee ATC (2015) | Inclusion: (a) aged 65 years and older, ethnic Chinese (b) living in the community Exclusion: (a) living in care homes (b) having history of stroke, Parkinson disease, or clinical dementia (c) scoring below the education-specific cutoff on C-MMSE at baseline (d) not providing a full description of their habitual exercise pattern | age, gender, education, medical and psychiatric history (hypertension, type 2 diabetes mellitus, hyper cholesterolemia, heart diseases, depression, stroke, Parkinson disease, and dementia), and lifestyle patterns (smoking and drinking) | ≥2 | ≥3 |
| Lee Y (2015) | Inclusion:  aged 65 years or older Exclusion: (a) proxy response on the K-MMSE (b) with cognitive impairment at baseline | N/A | 2 | 2 |
| Mao (2020) | Inclusion: (a) aged 80 years or older (b) with free cognitive impairment Exclusion: had severe cognitive impairment (MMSE score < 18) at baseline | age, gender, education, BMI, living pattern residence, current marital status, lifestyle behaviors such as smoking status, alcohol consumption, regular exercise, regular fresh fruit consumption, and vegetable consumption, prevalence of diabetes mellitus, cerebro vascular disease, and heart disease, activities of  daily living, and housework | 5.5 | 2.8 |
| Ogino (2019) | Inclusion: (a) community residents (b) aged 65 or older (c) fluent in English or Spanish (d) had no report of a dementia diagnosis or serious memory complaints | age, gender, education, ethnicity, BMI, smoking status, alcohol drinking, APOE ε4 genotype, comorbidities ( psychiatric diseases, diabetes, insulin treatment, heart disease, hypertension, head injury, and depression), self-reported occupation | 2-3 | 10 |
| Osuka (2020) | Exclusion: (a) had a disability in basic activities of daily living, neurological disease, or dementia, as diagnosed by physicians at the baseline survey (b) had a cognitive impairment assessed as <24 points on the MMSE at the baseline survey (c) had missing variables for the MMSE at the follow-up survey | age, BMI, education, number of cohabitants, medical history (participants whether they had physician-diagnosed hypertension, heart disease, diabetes, hyperlipidemia, or osteoporosis in the past year), depressive symptoms, and lifestyle factors (smoking and drinking history) | 2 | 2 |
| Qiu (2019) | Inclusion: (a) aged >80 years (b) free of cognitive impairment at 1998 Exclusion: with cerebrovascular disease, Parkinson’s disease, or totally limited physical function | age, gender, marital status, occupation, smoking, drinking, vegetarian, diabetes and depression | 3 | 2.1 |
| Sato (2021) | Inclusion: (a) aged 65 or older (b) physically and cognitively independent (i.e., not certifed as needing assistance from public long-term care insurance [LTCI]) Exclusion:  with dementia at baseline | gender, age, education, annual equivalized household income, marital status, paid work, the existence of heart disease, stroke, and diabetes, depressive symptoms, drinking habits, smoking status, family support, contact with friends, participation in community groups, population density, average degree of slopes, annual hours of sunlight, and fxed efects of municipalities | 2 | 5.7 |
| Yoon (2021) | Inclusion: (a) aged 65 years or older without preexisting dementia (b) had available health checkup data from the Korean National Health Insurance Service database | age, gender, BMI, Hospital Frailty Risk score, income, smoking, alcohol, hypertension, diabetes mellitus, dyslipidemia, chronic kidney disease, heart failure, vascular disease, prior ischemic stroke or transient ischemic attack, chronic obstructive pulmonary disease, and malignancy | 2-5 | 1 |
| Zhang (2021) | Exclusion: had severe cognitive impairment (MMSE score < 18) at baseline | gender, age, BMI, education, occupation before retirement, marital status, smoking, alcohol drinking, diversity of dietary protein intake, hypertension, diabetes, heart disease, and cerebrovascular disease | 3 | 3 |
| Zhou (2017) | Inclusion:  (a) aged ≥65 years  (b) free of dementia in 2002 | age, rural/urban residency, living alone, education level and main occupation before the age of 60 years, lifestyle factors (current smoking, current drinking, engaging in regular organised social activities, gardening, playing cards/mah-jong or listening to radio/watching television), health status (stroke or cerebrovascular disease, hypertension or diabetes) | 4 | 3 |
| Zhu (2017) | Inclusion: (a) aged ≥65 years  (b) free of cognitive impairment in 2002 | age, gender, education, occupation, residence, physical exercise, smoking, drinking, cardiovascular diseases and risk factors, negative well-being, and physical functioning, and baseline MMSE score | 2.7 | 3 |

APOE: Apolipoprotein E; BMI: body mass index
